# Cerebral Blood Flow and Cognitive Functioning in a Community-Based, Multi-Ethnic Cohort: The SABRE Study

**DOI:** 10.3389/fnagi.2018.00279

**Published:** 2018-09-18

**Authors:** Anna E. Leeuwis, Lorna A. Smith, Andrew Melbourne, Alun D. Hughes, Marcus Richards, Niels D. Prins, Magdalena Sokolska, David Atkinson, Therese Tillin, Hans R. Jäger, Nish Chaturvedi, Wiesje M. van der Flier, Frederik Barkhof

**Affiliations:** ^1^Department of Neurology, Amsterdam Neuroscience, Alzheimer Center Amsterdam, Vrije Universiteit Amsterdam Amsterdam UMC, Amsterdam, Netherlands; ^2^Department of Population Science and Experimental Medicine, Institute of Cardiovascular Science University College London, London, United Kingdom; ^3^Translational Imaging Group, Department of Medical Physics and Biomedical Engineering University College London, London, United Kingdom; ^4^MRC Unit for Lifelong Health and Ageing University College London, London, United Kingdom; ^5^Department of Medical Physics and Biomedical Engineering University College London, London, United Kingdom; ^6^Centre for Medical Imaging University College London, London, United Kingdom; ^7^Neuroradiological Academic Unit, Department of Brain Repair and Rehabilitation UCL Institute of Neurology, London, United Kingdom; ^8^Department of Epidemiology and Biostatistics, Vrije Universiteit Amsterdam Amsterdam UMC, Amsterdam, Netherlands; ^9^Institutes of Neurology and Healthcare Engineering University College London, London, United Kingdom; ^10^Department of Radiology and Nuclear Medicine, Vrije Universiteit Amsterdam Amsterdam UMC, Amsterdam, Netherlands

**Keywords:** cerebral perfusion, arterial spin labeling, cognition, neuropsychology, vascular risk factors, ethnicity

## Abstract

**Introduction:** Lower cerebral blood flow (CBF) is associated with cardiovascular disease and vascular risk factors, and is increasingly acknowledged as an important contributor to cognitive decline and dementia. In this cross-sectional study, we examined the association between CBF and cognitive functioning in a community-based, multi-ethnic cohort.

**Methods:** From the SABRE (Southall and Brent Revisited) study, we included 214 European, 151 South Asian and 87 African Caribbean participants (71 ± 5 years; 39%F). We used 3T pseudo-continuous arterial spin labeling to estimate whole-brain, hematocrit corrected CBF. We measured global cognition and three cognitive domains (memory, executive functioning/attention and language) with a neuropsychological test battery. Associations were investigated using linear regression analyses, adjusted for demographic variables, vascular risk factors and MRI measures.

**Results:** Across groups, we found an association between higher CBF and better performance on executive functioning/attention (standardized ß [stß] = 0.11, *p* < 0.05). Stratification for ethnicity showed associations between higher CBF and better performance on memory and executive functioning/attention in the white European group (stß = 0.14; *p* < 0.05 and stß = 0.18; *p* < 0.01 respectively), associations were weaker in the South Asian and African Caribbean groups.

**Conclusions:** In a multi-ethnic community-based cohort we showed modest associations between CBF and cognitive functioning. In particular, we found an association between higher CBF and better performance on executive functioning/attention and memory in the white European group. The observations are consistent with the proposed role of cerebral hemodynamics in cognitive decline.

## Introduction

Hemodynamic abnormalities, such as lower cerebral blood flow (CBF), are associated with cardiovascular disease and vascular risk factors, and are increasingly acknowledged as an important contributor to cognitive decline and dementia (Ott et al., 1999; Whitmer et al., 2005; Binnewijzend et al., 2013; Bangen et al., 2014; Wolters et al., 2016; Leeuwis et al., 2017). Earlier studies have shown that cerebral blood flow (CBF) is lower in patients with cognitive impairment or dementia, compared to healthy controls (Binnewijzend et al., 2013, 2015). CBF mapping can be accomplished with a non-invasive MRI-technique, arterial spin labeling (ASL), which uses magnetically labeled water as a tracer for blood flow. In a recent study, we found that lower ASL-measured CBF in mild cognitive impairment (MCI) and Alzheimer's disease (AD) was associated with worse performance on the Mini-Mental State Examination (MMSE) (Binnewijzend et al., 2013). In addition, we found that lower CBF was related to impairment in multiple cognitive domains in AD, suggesting CBF as potential functional marker of disease severity (Leeuwis et al., 2017). However, former studies on the association between CBF and cognitive functioning have been limited to cohorts with European-origin participants. Large differences in the incidence and prevalence of vascular risk profiles are recognized in ethnic minorities (Anand et al., 2000; Kurian and Cardarelli, 2007; Gijsberts et al., 2015), but little is known about how this affects the association between CBF and cognitive functioning. In the present study, we investigated the association between ASL-measured CBF with performance in global cognition and the cognitive domains of memory, executive functioning/attention and language in a multi-ethnic community-based cohort with white European, South Asian and African Caribbean participants.

## Methods

### Participants

**Figure 1:**
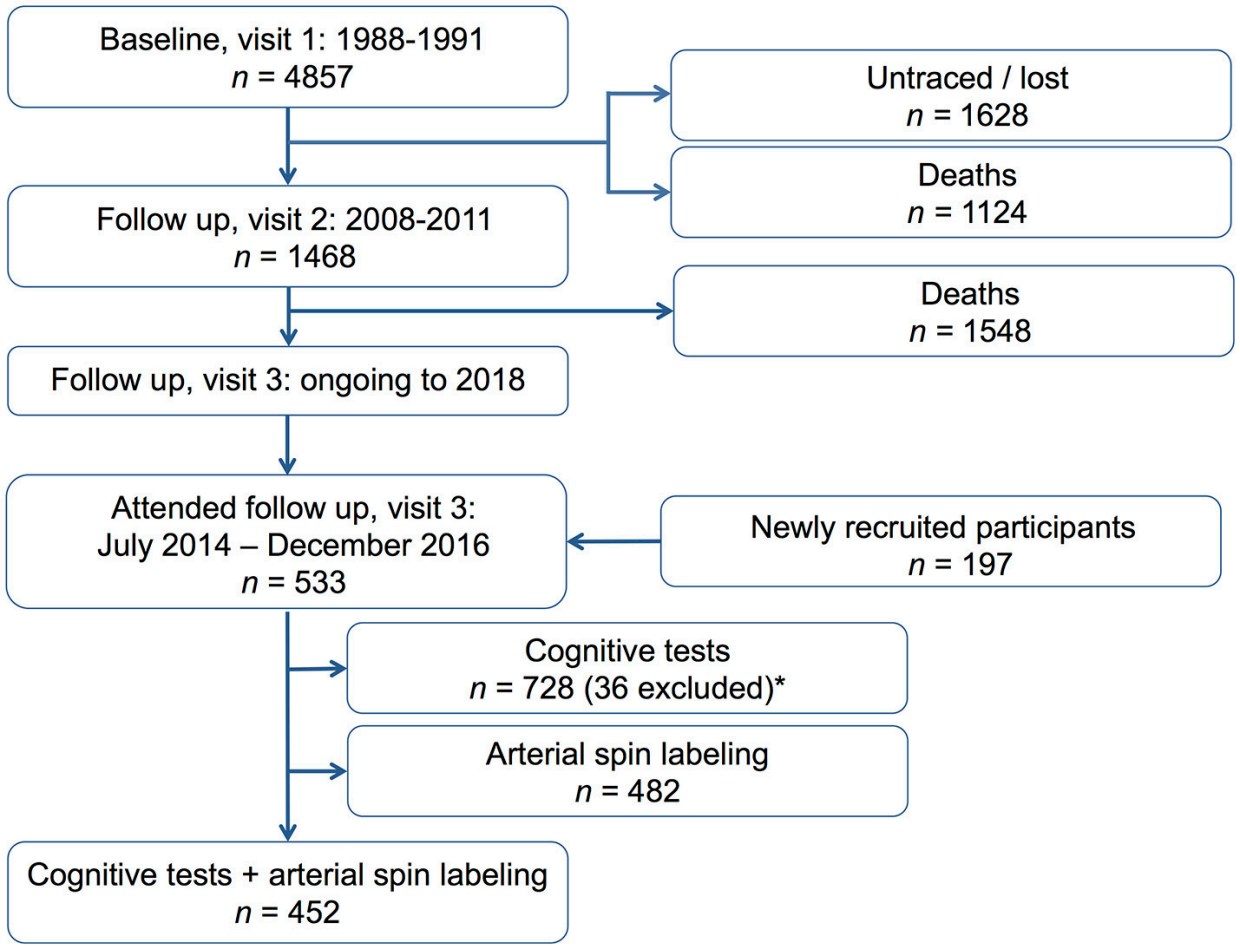
Flow diagram, showing our study population. *We excluded 36 participants with non-reliable testing results on neuropsychological assessment, due to literacy problems, impaired hearing, or impaired eyesight.

We analyzed follow-up data from the Southall And Brent Revisited (SABRE) study, a multi-ethnic community-based cohort consisting of white European, first-generation migrant South Asian, and African Caribbean men and women (Tillin et al., 2012). The SABRE study is a longitudinal study principally investigating cardio-metabolic disease. Index participants were recruited from primary care between 1988 and 1991, when they were aged 40–69 years. 4857 participants took part in the original study (2346 European, 1710 South Asian, 801 African Caribbean). Surviving participants were invited to attend the 20-year follow-up investigation between 2008 and 2011, and the 25-year follow-up investigation, which started in 2014 and was finished in January 2018. One thousand four hundred and thirty-eight participants attended for clinical follow-up in the 20-year follow-up investigation (2008–2011). At the 25-year follow-up investigation (between July 2014 and December 2016), 533 index participants attended the clinical visit [including 197 newly recruited participants (i.e., partners of index participants and new African-Caribbean participants)]. Figure 1 is a flow diagram showing the study population. Participants with available ASL and neuropsychological assessment during the first phase (July 2014–Dec 2016) of the 25-year follow-up investigation were included in this cross-sectional analysis. Participants with non-reliable testing results on neuropsychological assessment, due to literacy problems, or impaired hearing or eyesight were excluded. The current study included 452 participants (214 European, 151 South Asian, 87 African Caribbean). Approval was obtained from Fulham research ethics committee (ref:07/H0712/19) and all participants gave written informed consent.

### Clinical measurements

Participants had a light breakfast at home (tea or coffee limited to one cup) and refrained from alcohol and smoking on the morning of attendance, but took their usual medications on the clinic morning. Participants completed a questionnaire detailing sociodemographic characteristics (including years of education), health behaviors (including smoking status), medical history, and medication. Height, weight, and waist circumference were measured (Tillin et al., 2012). Hypertension was defined as physician diagnosed hypertension or use of blood pressure-lowering medication from participants' questionnaire and general practitioners' medical record review. Diabetes mellitus was defined as physician diagnosed diabetes or according to the World Health Organization 1999 guidelines (World Health Organization, 1999). Hematocrit (HCT) was measured using an impedance based, direct current sheath flow method (Sysmex XE-2100, Sysmex, Kobe, Japan) from a venous blood sample drawn on the same morning as the MRI examination.

### Neuropsychological assessment

We assessed cognitive functioning with a standardized neuropsychological test battery. The battery was assessed early in the clinic day attendance, to avoid fatigue. The battery comprised tests previously validated for cross-cultural settings (Stewart et al., 2001, 2003). We assessed global cognition and the three cognitive domains of memory, executive functioning/attention and language. For global cognition, we used the Community Screening Instrument for Dementia (CSI-D) (Hall et al., 2000; Prince et al., 2011). To generate a composite global score for the CSI-D, a standard algorithm was applied (Sosa et al., 2009). For memory, we used the 10-word total immediate recall and delayed recall of the Consortium to Establish a Registry for Alzheimer's disease (CERAD) (Fillenbaum et al., 1996). We combined the cognitive domains executive functioning and attention into one cognitive domain, using the forward and backward condition of the Digit Span and the Color Trails part A and B (Wechsler, 1997; Dugbartey et al., 2000; Lee et al., 2000). To assess language, we used the category fluency (animals) and the Boston Naming test (Goodglass and Kaplan, 1983). Raw neuropsychological test scores were standardized into *z*-scores. Color Trails A and B were log-transformed due to non-normal distribution and inverted by computing−1^*^z-score, so that higher scores imply better performance. Subsequently, available test scores were averaged to create the cognitive domains.

### MRI protocol

Participants underwent MRI on the same day as all the other examinations. MRI was performed on a Philips Achieva 3T scanner (Philips, Best, the Netherlands) using an 8-channel head coil. The MRI protocol included a sagittal T1-weighted, T2-weighted, gradient-echo T2^*^-weighted, diffusion weighted images (DWI) and fluid-attenuated inversion recovery (FLAIR) sequences. Structural MRI images were performed in the same scanning sessions as transversal 2D pseudo-continuous ASL (pcASL) perfusion images (EPI, TR/TE 4615/15 ms, flip-angle 90°, voxel size 3.75 × 3.75 × 5 mm), 1 mm slice gap, 20 slices, labeling duration 1,800 ms, post labeling delay 2,000 ms, 35 dynamics). Planning was aligned to the anterior commissure-posterior commissure line in the transversal plane orthogonal to the T1w, ensuring coverage of the entire cerebrum including the vertex.

### Brain and lesion volumes

An automated segmentation protocol (Geodesic Information Flows [GIF]) for T1-weighted images was used to yield regional volumes (Cardoso et al., 2015). GIF is part of the NiftySeg (http://niftyseg.sf.net) software package and is available at NiftyWeb (http://cmictig.cs.ucl.ac.uk/niftyweb). A multi-atlas segmentation propagation and fusion technique, Similarity and Truth Estimation for Propagated Segmentations (STEPS), was used to segment in brain structures (Jorge Cardoso et al., 2013). Hippocampal volumes were calculated as the sum of right and left segmented volumes. An automated segmentation method was used to define volumes and distribution of white matter hyperintensities (WMH). This method is also available at NiftyWeb.

### Preprocessing and MRI data analysis of ASL

Tissue segmentation and region labels were obtained using the Geodesic Information Flows framework (Cardoso et al., 2015). This method produces a state-of-the-art segmentation and regional labeling by voxel-wise voting between several propagated atlases, guided by the local image similarity. Gray matter and white matter were defined within the propagated atlases. Segmentations of the gray matter, white matter and cerebrospinal fluid (CSF) space were resampled to the space of the ASL acquisition making use of the known point-spread function to account for down-sampling induced loss of information. Determination of CBF maps from ASL data follows the simple derived form for pcASL (Equation 1) from the ISMRM recommendations white paper (Alsop et al., 2014) presented in units of ml/100 g/min and fitted with an open-source software package (Melbourne et al., 2016).

(1) $$\begin{array}{l} \text{CBF=6000}\lambda {\text{e}}^{\text{PLD/T1blood}}(\text{SC-SL})\text{/SPD}(\text{2}\alpha {\text{T1}}_{\text{blood}}\\ \;({\text{1-e}}^{\text{-}\tau \text{/T1blood}}))[\text{ml/100g/min}] \end{array}$$

Acquisition proceeded by acquiring a number of pairs of control (SC) and label (SL) data. These pairs were averaged to generate single voxel values for the control and label in (Equation 1) where λ is the plasma/tissue partition coefficient (0.9 ml/g), PLD the post-labeling delay between end of bolus and start of imaging, T1_blood_ the blood T1 value (1,650 ms), SPD the proton density, α the labeling efficiency (85%) and τ the labeling pulse duration. Partial volume correction (PVC) and T1 correction were applied (Asllani et al., 2008) where the T1 was adapted for HCT using the formula T1 = (0.52^*^HCT+0.38)^−1^ (Lu et al., 2004). The standard value for blood T1 at 3T is 1,650 ms, corresponding to a default HCT value of about 43%. We used the HCT value measured from each participant to calculate blood T1.

### Statistical analysis

Statistical analyses were performed using SPSS 22.0 for Mac (SPSS Inc., Chicago, IL). Participant characteristics for continuous data are reported as mean ± SD, categorical data are reported as number (percentage). We performed linear regression analyses to investigate associations between whole-brain CBF and cognitive functioning. We used CBF with correction for individual HCT as the independent variable and cognitive domains as the dependent variables. We included age, sex, education and ethnicity as covariates. In addition, we adjusted for variables that impact CBF and cognitive functioning in older adults: vascular risk factors (hypertension, diabetes and smoking) and MRI markers (WMH volume and hippocampal volume). First, we investigated the association between CBF with cognitive functioning, adjusted for age and sex (Model 1). We re-ran this model while one by one adding each of the other determinants to the model (Models 2–8). Finally, we entered all variables simultaneously (Model 9). Results were subsequently stratified for ethnicity. We provide standardized beta's (stß) to allow comparison of effect sizes.

## Results

### Participant characteristics

Participant characteristics and neuropsychological test results are shown in Table 1. The mean age of participants was 71 ± 5 years and 174 (38%) were female. White Europeans represented 47% of the sample, South Asians 33% and African Caribbean 19%. Hypertension was highly prevalent (58%), while 23% had diabetes mellitus. On average, participants performed within the normal range on neuropsychological tests.

**Table 1:** Clinical characteristics of participants.

**Characteristics**	**Total (*n* = 452)**
Women, *n* (%)	174 (38.5%)
Ethnicity, *n* (%)	
White European	214 (47%)
South Asian	151 (33%)
African Caribbean	87 (19%)
Age	71 ± 5
Years of education^§^	12 ± 3
Hypertension, *n* (%)^†^	262 (58%)
Diabetes mellitus, *n* (%)^†^	105 (23%)
Smoking, *n* (%)^†^^§^	
Never	149 (33%)
Ever	226 (50%)
**MRI-characteristics**	
Left + right hippocampal volume, mL^§^	7.1 ± 0.7
Total brain volume, mL	463.4 ± 45.4
Total white matter lesion volume in mL, median (IQR)^§^	5.8 (15.5)
Whole-brain HCT CBF^¥^	36.7 ± 6.3
**Cognitive test scores**	
Global cognition (*z*-score)	0.0 ± 0.7
CSI-D	30.4 ± 1.7
Memory (*z*-score)	0.0 ± 0.8
CERAD total immediate	18.5 ± 3.9
CERAD delayed	5.7 ± 1.9
Executive functioning/attention (*z*-score)	0.0 ± 0.7
Digit span (forward)	6.2 ± 1.2
Digit span (backward)	4.1 ± 1.3
Color trails, part A*	68.7 ± 37
Color trials, part B*	148.9 ± 66.9
Language (*z*-score)	0.0 ± 0.7
Animal fluency	18.9 ± 6.6
Boston naming test^¶^	8.6 ± 0.6

*MRI, magnetic resonance imaging; CBF, cerebral blood flow; HCT, hematocrit level correction; IQR, interquartile range; CSI-D, community screening instrument for dementia; CERAD, consortium to establish a registry for Alzheimer's disease*.

† *History of hypertension, diabetes mellitus and smoking status was determined based on self-reported medical history and/or medication use*.

§ *Missing values: years of education: 33/452; smoking status 77/452; hippocampal volume 2/452; white matter lesion volume 27/452*.

¥ *CBF-values in mL/100 g/min*.

* *Higher scores imply worse performance*.

¶ *Boston Naming Test range: 0–9*.

*Data are presented as mean ± SD or number (percentage). z-scores allow comparison of neuropsychological test results within participants. Higher z-scores imply better performance on all tests*.

### Association between cerebral blood flow and cognitive functioning

Table 2 shows the association between CBF and cognitive functioning, adjusted for age and sex (Model 1). Next, we re-ran this analysis with additional adjustment for each of the putative confounders in a separate model (Models 2–8). Adjusted for age and sex (Model 1), we found associations between CBF and each of the cognitive domains (standardized beta [stß] = 0.14–0.18, *p* < 0.01). Subsequent adjustment for covariates in Model 2–8 showed that—with exception of ethnicity—none of the covariates confounded this association. When we finally entered all variables simultaneously in a multivariate model, we found that the association between CBF and performance on executive functioning/attention remained significant (stß = 0.11, *p* < 0.05). We found no association between CBF and global cognition, memory or language. Subsequent stratification for ethnicity showed that this association was largely attributable to the white European subgroup. In this group, we found associations between higher CBF and better performance on executive functioning/attention (stß = 0.18, *p* < 0.01) and an association between CBF and memory (stß = 0.14, *p* < 0.05). We found much weaker associations between CBF and cognitive functioning in the South Asian and African Caribbean group. Subsequently, we repeated all analyses with partial volume corrected (PVC) CBF. These analyses showed a similar pattern of associations (data not shown).

**Table 2:** Linear regression models for the association between CBF and cognitive functioning.

**Group**	**Cognitive domain**	**Model**								
		**1**	**2**	**3**	**4**	**5**	**6**	**7**	**8**	**9**
		**Age and sex**	**Model 1 + education**	**Model 1 + ethnicity**	**Model 1 + hyper-tension**	**Model 1 + diabetes**	**Model 1 + smoking**	**Model 1 + WMH volume**	**Model 1 + hippo-campal volume**	**Full model (all variables)**
All	Global cognition	0.18**	0.18***	0.07	0.15**	0.16**	0.15**	0.17**	0.16**	0.06
(*n* = 452)	Memory	0.15***	0.14**	0.11*	0.14**	0.14**	0.12*	0.15*	0.14**	0.06
	Executive func/attention	0.17***	0.16**	0.09*	0.15*	0.15**	0.18***	0.18***	0.16**	0.11*
	Language	0.14**	0.13*	0.03	0.12*	0.13**	0.10	0.13**	0.13**	−0.02
European	Global cognition	0.12	0.11	*n*.*a*.	0.11	0.11	0.13	0.12	0.11	0.14
(*n* = 215)	Memory	0.13*	0.12*	*n*.*a*.	0.13*	0.13*	0.13*	0.14*	0.13*	0.14*
	Executive fun /attention	0.18**	0.18**	*n*.*a*.	0.18**	0.18**	0.19**	0.17*	0.18**	0.18**
	Language	0.01	0.00	*n*.*a*.	0.01	0.01	0.01	−0.01	0.00	−0.02
South Asian	Global cognition	0.04	0.06	*n*.*a*.	0.03	0.04	−0.01	0.04	0.04	0.00
(*n* = 151)	Memory	0.07	0.07	*n*.*a*.	0.05	0.07	−0.06	0.08	0.07	−0.05
	Executive func/attention	−0.01	−0.04	*n*.*a*.	−0.03	−0.01	0.02	0.05	−0.01	−0.01
	Language	−0.00	0.01	*n*.*a*.	−0.01	−0.00	−0.04	−0.02	0.01	−0.07
African Caribbean	Global cognition	0.04	0.04	*n*.*a*.	0.01	0.01	0.04	−0.06	0.05	−0.11
(*n* = 87)	Memory	0.05	0.02	*n*.*a*.	0.04	0.02	−0.02	−0.01	0.04	−0.15
	Executive func/attention	0.04	−0.01	*n*.*a*.	0.02	0.04	0.10	0.02	0.05	0.05
	Language	−0.10	0.10	*n*.*a*.	0.08	0.07	0.03	0.10	0.10	0.02

*Executive func, executive functioning; WMH, white matter hyperintensities; n.a., not applicable*.

* *p < 0.05*,

** *p < 0.01*,

*** *p < 0.001*.

*Results are presented as standardized beta [stß] to allow comparison of effect sizes. We used linear regression analyses with cerebral blood flow (CBF) as independent variable and cognitive domains as dependent variable. Cognition is expressed as (composite) z-score. Model 1: Univariate regression analysis, adjusted for age and sex; Model 2–8: All analyses are adjusted for age and sex + named variable; Model 9: We entered all variables (age, sex, education, ethnicity, vascular risk factors and MRI measures) simultaneously in one model (Enter method). Results were subsequently stratified for ethnicity*.

## Discussion

In a multi-ethnic community-based cohort we found modest associations between CBF and cognitive functioning. In particular, we found an association between higher CBF and better performance on executive functioning/attention and memory in the white European group.

Our findings are in line with earlier studies examining CBF and cognition. For example, the Rotterdam Study investigated the relationship between CBF using 2D phase-contrast MRI and cognitive functioning and found small effect sizes for information processing speed and executive functioning (difference in Z-score per SD increase in flow measure: 0.04 (CI: −0.02; −0.09) and 0.00 (CI: −0.05; 0.05) respectively) (Poels et al., 2008). In addition, a study of the association between CBF and cognitive decline found a modest effect size on lower CBF and accelerated decline in global cognition (ß = −0.029) (Wolters et al., 2017).

Previously, we investigated the association between CBF and cognitive functioning in a memory clinic cohort and we found effect sizes comparable to the current study (stß = 0.09–0.14, all *p* < 0.05). Stratification for syndrome diagnosis (cognitively normal, MCI and dementia) showed that this association was mainly attributable to the dementia group and was least obvious in the cognitively normal group (stß = 0.01–0.07, all *p* > 0.05). These findings support the potential role of ASL as measure of disease severity, as we found only an association in the dementia group. In the current study we did find a significant albeit modest association between CBF and cognition in a community-based population. We previously investigated CBF in the predementia phase of AD and we found that CBF alterations occur further along the disease process of AD and were only reduced in more advanced stages of AD (Binnewijzend et al., 2015). In combination with the current results, this would suggest that changes in ASL-measured CBF are less sensitive in the early stages of cognitive impairment and dementia. In addition, our modest results might be attributable to the comparatively high prevalence of adverse vascular risk factors in this multi-ethnic study of older adults. Earlier studies suggest that reduced CBF is a major factor in the development of vascular cognitive impairment (VCI) (Sabayan et al., 2012; Yang et al., 2017). In a former study we showed that decreased CBF in a memory clinic cohort not only reflects disease burden of neurodegeneration, but is also related to small vessel disease (SVD) and (cerebro-)vascular factors (Benedictus et al., 2014).

The most prominent finding was the association between CBF and executive functioning/attention. This is consistent with the observation that executive dysfunction is common in patients with VCI(Prins and Scheltens, 2015). In addition, we also found an association with memory. However, although cognitive tests are selected to measure a specific cognitive domain, most tests involve multiple cognitive processes. For example, the CERAD (verbal memory test) not only measures memory but also requires attention and executive control in list organization. Consistent with our findings, effect sizes of the relations between vascular brain injury and cognitive functioning in cognitively normal elderly are generally modest. For example, a recent meta-analysis on the association between WMH and cognitive functioning showed small effect sizes for memory and executive functioning/attention (Fisher z-score −0.08 [CI: −0.13; −0.06] and −0.11 [CI: −0.16; −0.07] respectively) (Kloppenborg et al., 2014). In addition, a recent meta-analysis on the effect of microbleeds showed a small negative association with global cognitive functioning (individuals with microbleeds −0.3 MMSE point lower than those without; Li et al., 2017).

Among the limitations of the study is its cross-sectional design, which prevents us from drawing conclusions about causality. We cannot exclude potential survival bias as the least healthy participants were less likely to participate in the 25-year follow-up study, used for the current study. Second, we found that common vascular risk factors do not confound the associations between CBF and cognition, while ethnicity acted as a confounder. It is known that vascular risk factors increase the risk of cognitive impairment and are associated with reduced CBF (Gorelick et al., 2011; Muller et al., 2012). Reduced CBF has been associated with poorer cognitive performance in older patients with high vascular risk, suggesting that older adults with multiple risk factors may be particularly vulnerable to cognitive changes as a function of reduced CBF (Bangen et al., 2014). Contrary to our expectation, our results could not be attributed to South Asian and African Caribbean participants having higher burden of vascular risk factors, although there may have been other group differences not accounted for in our analyses.

In addition, we did not adjust for possible confounders such as caffeine-intake and vasoactive medication. The Rotterdam Study recently showed that hippocampal subregions, in particular the subiculum, were associated with cognitive functioning, over and above that of total hippocampal volume (Evans et al., 2018). Our study showed that total hippocampal volume did not confound the association between CBF and cognitive functioning. Unfortunately, we did not have information on hippocampal subregions in this study. The relatively small sample size of the South Asian and African Caribbean subgroups could indicate lack of adequate statistical power to reveal associations. Alternatively, the neuropsychological test battery which has been validated for cross-cultural settings (Nguyen et al., 2007; Robertson et al., 2007) may not have been sensitive enough to detect specific or subtle cognitive deficits in this community-based cohort. Furthermore, literacy influences the specificity of neuropsychological measures but is also a strong factor in determining level of cognitive functioning. We excluded participants with obvious literacy problems from our analyses, but suboptimal literacy and numeracy in our cohort could have influenced our results.

The main strengths of our study were the large ethnically diverse community-based cohort, the use of a standardized ASL-MRI protocol and a standardized neuropsychological test battery. We used individualized HCT adjusted CBF estimates. A recent study in this cohort showed that HCT levels differed according to sex and ethnicity and that this influenced the CBF estimates (Smith et al., under review). In our study, analyses with adjustment for partial volume yielded comparable results, confirming that our findings were not confounded by cerebral atrophy.

In summary, we have shown modest associations between CBF and cognitive functioning in a cohort of multi-ethnic elderly with a high prevalence of cardiovascular risk factors. These findings support the notion that CBF is less sensitive to early brain changes and has particular value in more advanced disease stages of dementia.

## Ethics statement

This study was carried out in accordance with the recommendations of the Fulham research ethics committee with written informed consent from all participants. All participants gave written informed consent in accordance with the Declaration of Helsinki. The protocol was approved by the Fulham research ethics committee.

## Author contributions

AL, NC, WF, and FB study concept and design. LS, AM, MS, DA, and TT acquisition of data. AL data analysis, data interpretation, preparation of manuscript. NC, WF, and FB made substantial contributions to the interpretation of the data and supervised the project. All authors: critical review, intellectual content of manuscript. All authors had final responsibility for the decision to submit for publication.

### Conflict of interest statement

The authors declare that the research was conducted in the absence of any commercial or financial relationships that could be construed as a potential conflict of interest. The handling editor declared a past co-authorship with one of the authors FB.

## Abbreviations

AD: Alzheimer's disease
ASL: arterial spin labeling
CBF: cerebral blood flow
HCT: hematocrit
MCI: mild cognitive impairment
MMSE: Mini-mental state examination
MRI: magnetic resonance imaging
SABRE: Southall And Brent Revisited
stß: standardized beta
VCI: vascular cognitive impairment
WMH: white matter hyperintensities.
